# Sex- and age-specific disparities in low back pain burden attributable to three modifiable risks, 1990–2021: A cross-sectional analysis of the global burden of disease study 2021

**DOI:** 10.1097/MD.0000000000051125

**Published:** 2026-11-06

**Authors:** Yulan Ba, Xuetao Yu, Erman Wu, Jinxian Li

**Affiliations:** a Department of Rehabilitation Medicine, People’s Hospital of Xinjiang Uygur Autonomous Region, Urumqi, China; b Department of Neurosurgery, the First Affiliated Hospital of Xinjiang Medical University, Urumqi, Xinjiang, China.

**Keywords:** body mass index, disability-adjusted life years, global health, low back pain, occupational exposure, smoking

## Abstract

Low back pain (LBP) is the leading cause of global disability, with more than 500 million cases reported in 2020, and its risk patterns vary across regions and demographic groups. Using Global Burden of Disease 2021 data, Disability-Adjusted Life Years (DALYs) attributable to 3 risk factors were analyzed across 204 countries. Age-standardized rates (ASRs) and temporal trends average annual percentage change (AAPC) were calculated. From 1990 to 2021, DALYs attributable to high body mass index (BMI) increased from 3.09 million to 8.36 million, while the ASR increased by 39%, disproportionately affecting high- sociodemographic index (SDI) regions (115.59 per 100,000 in 2021). ASRs attributable to occupational ergonomic factors and smoking declined by 19% and 33%, respectively, although absolute DALYs increased with population growth. Occupational ergonomic factors remained the leading contributor in 2021, particularly in low-income regions and Eastern Sub-Saharan Africa (ASR, 342.78 per 100,000). East Asia had the largest decline (AAPC = –1.43), whereas Oceania had the largest increase (AAPC = 1.07). Kiribati and Paraguay had the largest increases in occupational-related LBP, whereas Ethiopia and Moldova had the largest declines. Smoking-related DALYs declined in most regions but increased in Eastern Europe (AAPC = 0.18). Central Latin America and Southern Sub-Saharan Africa had the steepest declines (AAPC = –1.79 and –1.62, respectively). South Africa and Madagascar had the largest reductions in smoking-related LBP (AAPC = –1.94 and –2.05), whereas Uzbekistan and Mali had notable increases (AAPC = 0.70 and 1.00). The 50 to 54-year age group had the highest DALY burden across all 3 risk factors. However, DALYs attributable to high BMI peaked among females aged 70 to 74 years, whereas those attributable to occupational ergonomic factors peaked at 50 to 60 years and smoking-related DALYs at 60 to 69 years. Females had higher BMI- and occupation-attributable DALYs, whereas males had higher smoking-attributable burdens. SDI was strongly correlated with risk patterns: high BMI (*R* = 0.671) and smoking (*R* = 0.721) burdens increased with SDI, whereas occupational factors were inversely correlated with SDI (*r* = −0.414). The growing LBP burden is increasingly attributable to high BMI, particularly among aging populations and in high-SDI regions, whereas declining occupational and smoking risks obscure persistent disparities in low-income areas.

## 1. Introduction

Low back pain (LBP) is among the most prevalent musculoskeletal disorders globally and represents a significant burden on individuals and public health.^[1–3]^ The Global Burden of Disease (GBD) Study 2021 identified LBP as a leading cause of disability across demographic and occupational groups.^[4]^ Its multifactorial etiology includes several modifiable risk factors, notably high body mass index (BMI), occupational ergonomic factors, and smoking. Elevated BMI is consistently associated with increased LBP risk. Numerous studies suggest a direct correlation between excess weight and the incidence of LBP, where biomechanical stresses contribute to spinal degeneration and pain.^[5]^ GBD 2021 data indicate a substantial increase in musculoskeletal disorders, including LBP attributable to high BMI across populations.^[4]^ Evidence also supports weight management and obesity prevention as important strategies for preventing and alleviating LBP.^[6,7]^ Because high BMI increases lumbar spinal stress, public health interventions targeting obesity may reduce the incidence and burden of LBP.

Occupational ergonomic factors are another important contributor to LBP. Work-related musculoskeletal disorders are common in occupations involving physical labor, prolonged standing, or repetitive movements.^[8]^ Poorly designed work environments may increase physical strain, aggravate existing conditions, and contribute to new disorders.^[9]^ GBD data identify occupational factors as a major contributor to LBP burden, highlighting the need for ergonomic assessment and improved workplace design.^[10]^ Appropriate ergonomic practices may improve worker productivity while reducing the risk of chronic pain, including LBP.^[11]^

Smoking is also associated with LBP and chronic pain. Nicotine may adversely affect spinal health by impairing blood flow and delaying tissue healing.^[12]^ Studies have reported higher odds of LBP among smokers than nonsmokers.^[6,13]^ As a modifiable risk factor, smoking provides an opportunity for targeted interventions, including smoking cessation programs, to reduce LBP prevalence.^[14]^

This study examined the LBP burden attributable to 3 risk factors from 1990 to 2021 by sociodemographic index (SDI), age, and sex to characterize differences in burden patterns. Longitudinal and cross-sectional comparisons of risk exposure and attributable disease burden provide evidence to inform policies and interventions aimed at preventing and controlling the increasing global burden of LBP.

## 2. Materials and methods

### 2.1. Data source

Data on LBP burden attributable to risk factors were obtained from the Global Health Data Exchange through the GBD Results Tool (http://ghdx.healthdata.org/gbd-results-tool). Global Health Data Exchange is a public repository providing detailed findings from GBD studies conducted by the Institute for Health Metrics and Evaluation. This study used publicly available, de-identified, aggregate data from GBD 2021 and did not involve individual-level participant data; therefore, institutional review board approval and informed consent were not required. The GBD 2021 study assessed 88 risk factors across 204 countries and territories.^[4]^ Its hierarchical risk-factor framework evaluates individual risks and their aggregates through 6 analytical steps. Detailed information on the source data, methodology, and disease model for LBP in GBD 2021 has been reported previously.^[15,16]^

Data on LBP burden attributable to high BMI, occupational ergonomic factors, and smoking from 1990 to 2021 were collected, including corresponding 95% uncertainty intervals (UIs). Sex, age, SDI, and geographic location were also collected to characterize disparities in disease burden. Age-specific DALY rates attributable to each risk factor were additionally obtained. Estimates were reported as counts or age-standardized rate (ASRs) per 100,000 population, with corresponding 95% UIs. Uncertainty was propagated through 500 draws at each stage of the GBD estimation process. Point estimates represented the mean across draws, and 95% UIs were defined by the 2.5th and 97.5th percentiles of the draw distribution.

### 2.2. Case and exposure definitions

LBP was defined as pain, with or without referral to 1 or both lower limbs, lasting ≥1 day. The “low back” was defined as the posterior body region extending from the lower margin of the 12th ribs to the lower gluteal folds.^[17]^ The corresponding ICD codes were 724.2 (ICD–9) and M54.5 (ICD–0).

SDI is a widely used indicator of national socioeconomic development. The 204 countries and territories were categorized into 5 SDI quintiles (low, low-middle, middle, high-middle, and high) and 21 GBD regions.^[17,18]^

For adults aged ≥20 years, the GBD comparative risk assessment defined high BMI as exposure above the theoretical minimum risk exposure level of 20 to 23 kg/m^2^.^[4]^ This range represents the counterfactual minimum-risk exposure distribution used to estimate population-attributable burden and should not be interpreted as the World Health Organization (WHO) clinical threshold for overweight classification.^[19]^ For children and adolescents aged 2 to 19 years, overweight and obesity were defined using age- and sex-specific international BMI reference standards.^[20]^

Occupational ergonomic exposure was estimated from the proportion of the working population engaged in activities associated with increased LBP risk. Occupations were classified into 9 categories: managers; professionals; technicians and associate professionals; clerical support workers; service and sales workers; skilled agricultural, forestry, and fishery workers; craft and related trades workers; plant and machine operators and assemblers; and elementary occupations.^[16,21]^

Current smokers were defined as individuals who used any smoked tobacco product daily or occasionally. Former smokers were defined as individuals who had discontinued all smoked tobacco products for ≥6 months, where this information was available, or according to the definition used in the original survey.^[4,16]^

### 2.3. Statistical analysis

Disability-adjusted life years (DALYs) were analyzed by sex, age group, SDI quintile, GBD region, and country or territory. Temporal trends from 1990 to 2021 were assessed using the Joinpoint Regression Program (version 5.0.2), with the average annual percentage change (AAPC) calculated using default settings. The 95% confidence intervals (CIs) for AAPCs were derived from regression coefficients and their standard errors. An AAPC was considered statistically significant when its 95% CI excluded zero; a CI entirely above zero indicated an increasing trend, whereas one entirely below zero indicated a decreasing trend.

The association between socioeconomic development and LBP burden was assessed at the national level using a generalized linear model to examine the relationship between ASRs and SDI. Pearson’s correlation coefficient was used to assess the strength and statistical significance of the linear association. Joinpoint regression analyses were performed using the Joinpoint Regression Program, version 5.0.2. All other statistical analyses and data visualizations were performed using R software, version 4.3.1.

## 3. Result

### 3.1. Overall impact of risk factors on the global LBP burden

From 1990 to 2021, absolute DALYs attributable to all 3 risk factors increased, whereas age-standardized trends varied substantially. DALYs attributable to high BMI increased from 3.09 million (95% UI: 0.31–6.48 million) in 1990 to 8.36 million (95% UI: 0.84–17.42 million) in 2021, with the age-standardized DALY rate increasing from 70.22 to 97.66 per 100,000 population. DALYs attributable to occupational ergonomic factors increased from 10.85 million (95% UI: 7.60–14.54 million) to 15.57 million (95% UI: 11.03–20.91 million), whereas the corresponding ASR declined from 226.82 to 183.82 per 100,000 population. Similarly, smoking-attributable DALYs increased from 6.78 million (95% UI: 4.07–10.07 million)–8.82 million (95% UI: 5.18–13.13 million), while the ASR decreased from 153.22 to 102.04 per 100,000 population. In 2021, occupational ergonomic factors remained the largest contributor to absolute LBP DALYs, followed by smoking and high BMI (Tables S1, S2, S3, Supplemental Digital Content 1).

The age-standardized DALY rate attributable to high BMI increased, with an AAPC of 1.07 (95% CI: 1.06–1.09) (Table 1). In contrast, rates attributable to occupational ergonomic factors and smoking declined, with AAPCs of −0.68 (95% CI: −0.72−0.63) and −1.31 (95% CI: −1.35–− 1.27), respectively (Figure 1 and Table 1). Among the 3 risk factors, occupational ergonomic factors in females showed slower declines during 1993 to 2005 and 2019 to 2021, whereas the other factors demonstrated consistent linear trends over the 3 decades (Fig. 2).

**Table 1:** Annual average percentage change (AAPC) in age-standardized DALY rates of low back pain attributable to risk factors, 1990–2021 (95% CI).

Characteristics	High body-mass index	*P*-value	Occupational ergonomic factors	*P*-value	Smoking	*P*-value
Global	1.07 (1.06–1.09)	<.001	−0.68 (−0.72–−0.63)	<.001	−1.31 (−1.35 –−1.27)	<.001
Gender						
Female	1.03 (1.01–1.05)	<.001	−0.55 (−0.59–−0.51)	<.001	−1.56 (−1.6 −1.52)	<.001
Male	1.21 (1.2–1.23)	<.001	−0.82 (−0.91–−0.74)	<.001	−1.18 (−1.2 −1.15)	<.001
SDI						
Low SDI	1.93 (1.91–1.95)	<.001	−0.67 (−0.7–−0.64)	<.001	−1.03 (−1.06 −0.99)	<.001
Low-middle SDI	2.32 (2.31–2.34)	<.001	−0.44 (−0.5–−0.38)	<.001	−1.02 (−1.05 to −0.98)	<.001
Middle SDI	2.07 (2.06–2.09)	<.001	−1.11 (−1.17–−1.05)	<.001	−1.32 (−1.37 – −1.27)	<.001
High-middle SDI	0.74 (0.72–0.76)	<.001	−0.95 (−0.99–−0.9)	<.001	−0.83 (−0.85 – −0.81)	<.001
High SDI	1 (0.98–1.01)	<.001	−0.39 (−0.43– −0.35)	<.001	−1.13 (−1.15 – −1.11)	<.001
Region						
Australasia	1.18 (1.15–1.22)	<.001	−0.34 (−0.38–−0.31)	<.001	−1.21 (−1.25 – −1.17)	<.001
Oceania	0.82 (0.81–0.84)	<.001	1.07 (1–1.15)	<.001	−0.16 (−0.2 –−0.12)	<.001
East Asia	2.58 (2.52–2.63)	<.001	−1.43 (−1.51–−1.35)	<.001	−1.14 (−1.17 – −1.12)	<.001
Central Asia	0.77 (0.76–0.78)	<.001	−0.26 (−0.31–−0.21)	<.001	−0.05 (−0.09 – −0.02)	.001
South Asia	2.79 (2.74–2.84)	<.001	−0.97 (−1.01–−0.92)	<.001	−1.59 (−1.68– −1.51)	<.001
Southeast Asia	2.6 (2.56–2.63)	<.001	−0.19 (−0.24 –−0.14)	<.001	−0.58 (−0.59 – −0.56)	<.001
High-income Asia Pacific	1 (0.95–1.06)	<.001	−0.47 (−0.53–−0.41)	<.001	−1.21 (−1.23 – −1.19)	<.001
Eastern Europe	0.91 (0.89–0.92)	<.001	−0.46 (−0.51–−0.41)	<.001	0.18 (0.13–0.23)	<.001
Central Europe	0.78 (0.77–0.8)	<.001	−0.52 (−0.59–−0.45)	<.001	−0.49 (−0.5 −0.48)	<.001
Western Europe	1.04 (1.01–1.06)	<.001	0.01 (−0.04–0.06)	.684	−0.77 (−0.8 −0.75)	<.001
High-income North America	0.86 (0.81–0.91)	<.001	−0.41 (−0.46–−0.37)	<.001	−1.26 (−1.29 −1.23)	<.001
Andean Latin America	1.67 (1.65–1.69)	<.001	1 (0.92–1.08)	<.001	−0.35 (−0.37 −0.33)	<.001
Central Latin America	1.41 (1.4–1.42)	<.001	0.09 (0.06–0.11)	<.001	−1.47 (−1.55 −1.39)	<.001
Southern Latin America	1.22 (1.13–1.32)	<.001	0.4 (0.32–0.48)	<.001	−0.57 (−0.66 −0.48)	<.001
Tropical Latin America	1.62 (1.61–1.64)	<.001	−0.11 (−0.2–−0.02)	.021	−1.79 (−1.84 −1.75)	<.001
Caribbean	1.38 (1.37–1.39)	<.001	0.52 (0.44–0.6)	<.001	−1.01 (−1.04 −0.98)	<.001
North Africa and Middle East	1.74 (1.73–1.76)	<.001	−0.4 (−0.46–−0.34)	<.001	−0.48 (−0.52 −0.45)	<.001
Eastern Sub-Saharan Africa	2.08 (2.06–2.09)	<.001	−0.56 (−0.6–−0.51)	<.001	−0.76 (−0.8 −0.73)	<.001
Central Sub-Saharan Africa	2.77 (2.74–2.81)	<.001	−0.78 (−0.86–−0.7)	<.001	−0.41 (−0.46 −0.36)	<.001
Southern Sub-Saharan Africa	1.13 (1.12–1.14)	<.001	−0.17 (−0.23 –−0.1)	<.001	−1.62 (−1.65 −1.58)	<.001
Western Sub-Saharan Africa	1.82 (1.8–1.83)	<.001	−0.37 (−0.42–−0.32)	<.001	−0.83 (−0.87 −0.79)	<.001

**Figure 1.**
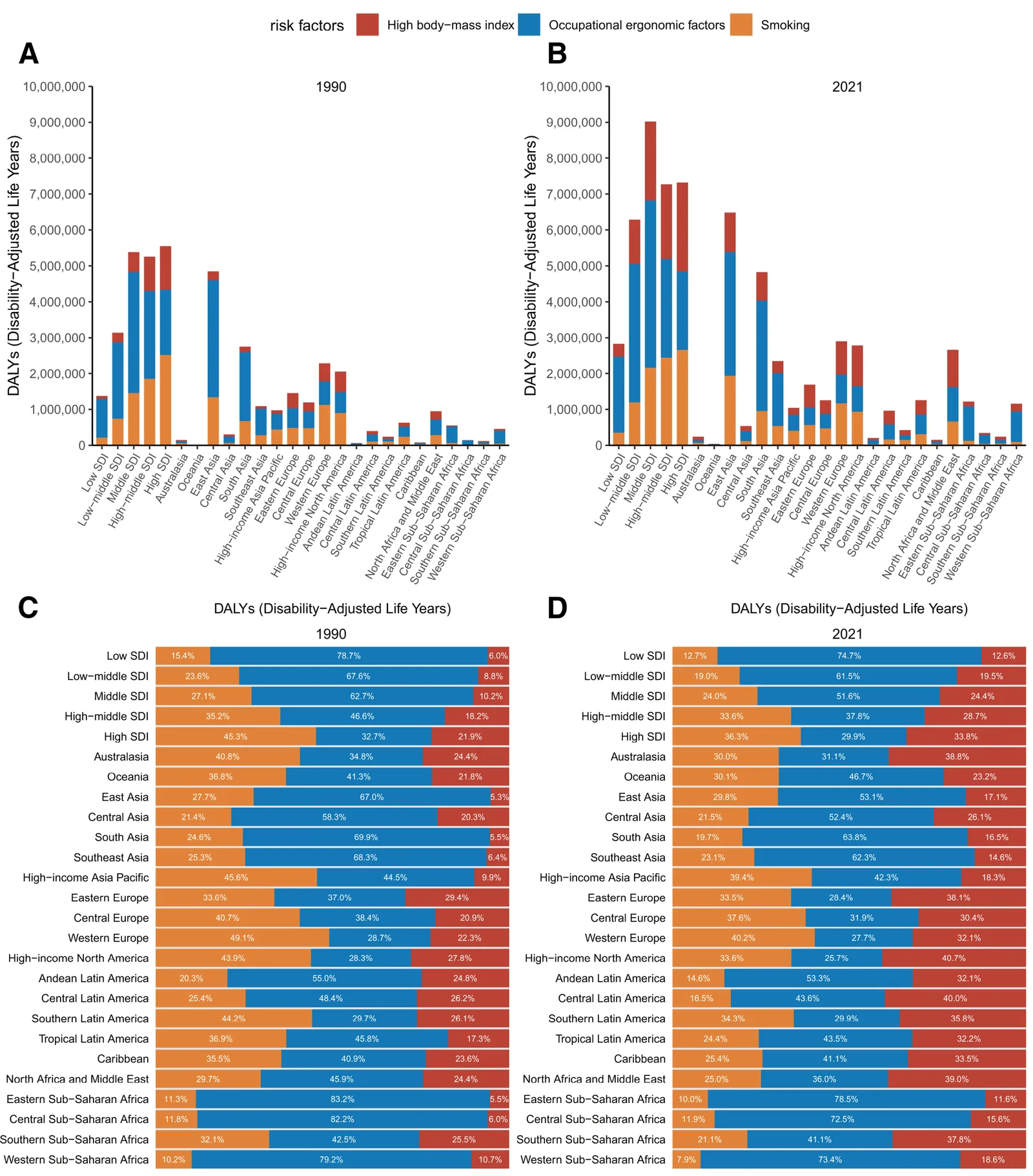
DALYs from low back pain attributable to risk factors across GBD and SDI regions in 1990 (a) and 2021 (b), with proportional distributions in 1990 (c) and 2021 (d). DALYs = disability-adjusted life years, GBD = global burden of disease, SDI = sociodemographic index.

**Figure 2.**
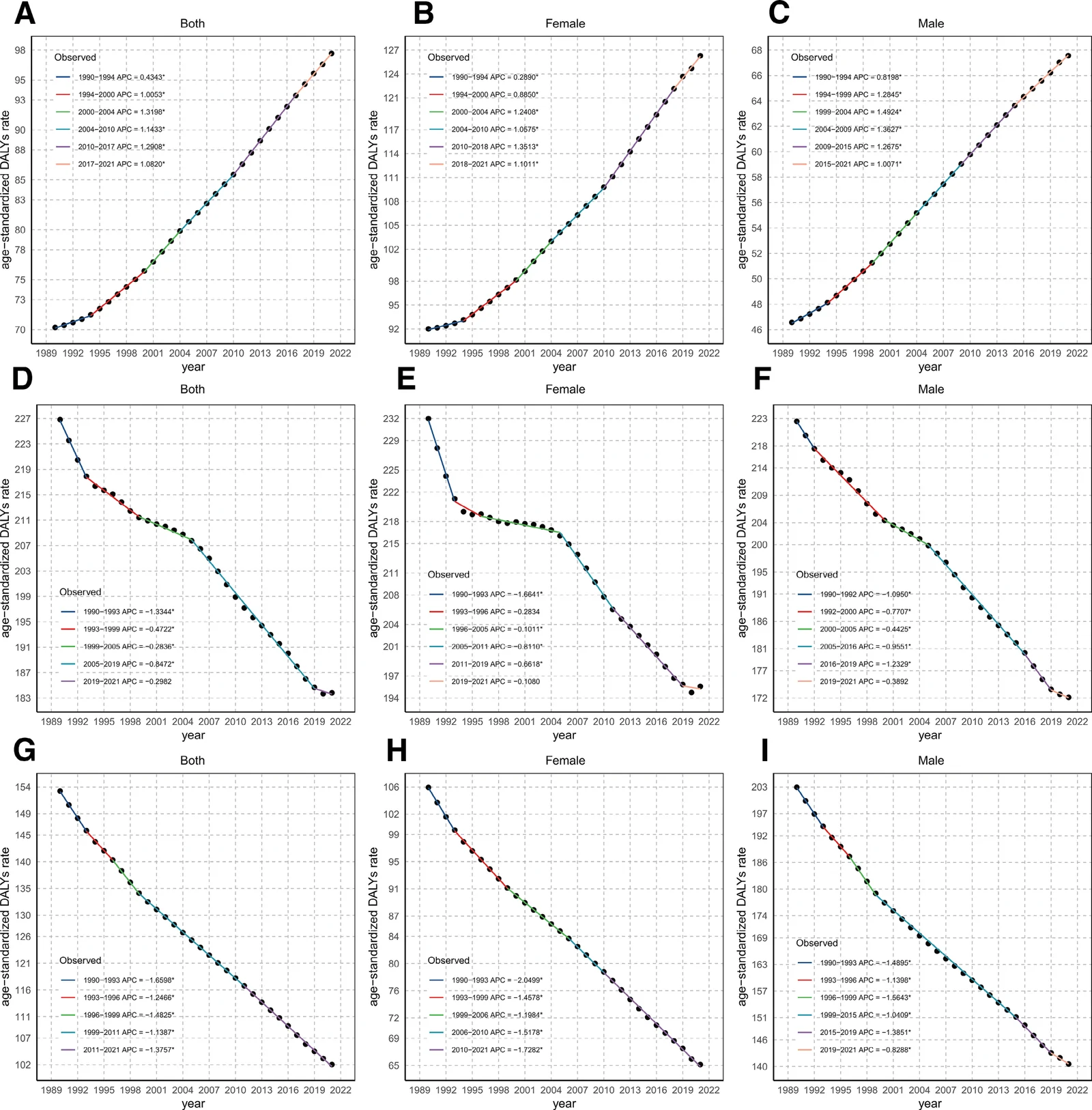
Sex-specific annual percentage change (APC) and trends in global low back pain attributable to high body mass index (panels A–C), occupational ergonomic factors (panels D–F), and smoking (panels G–I), with age-standardized DALY rates from 1990 to 2021. APC = annual percentage change, DALYs = disability-adjusted life years.

Across the 5 SDI regions, occupational ergonomic and smoking-related burdens declined, whereas the high BMI-attributable burden increased (Table 1). Low-middle SDI regions had the largest increase in high-BMI-attributable LBP DALYs (AAPC = 2.32; 95% CI: 2.31–2.34), whereas high-SDI regions had the highest absolute DALYs (2478,626.07; 95% UI: 249,211.24–5091,840.29) and age-standardized DALY rate (115.59; 95% UI: 11.61–238 per 100,000). The middle-SDI region had the largest declines in occupational ergonomic factors (AAPC = −1.11; 95% CI: −1.17– − 1.05) and smoking (AAPC = −1.32; 95% CI: −1.37– − 1.27) (Tables S2–S3, Supplemental Digital Content 2).

High BMI and smoking showed relatively consistent increasing and decreasing trends, respectively, across all 5 SDI regions (Fig. 3). In contrast, occupational ergonomic factors increased during specific periods: 1990 to 1995 in low-SDI regions annual percentage change ([APC] = 0.2419); 1990 to 1996 (APC = 0.5522) and 1999 to 2006 (APC = 0.3714) in low-middle-SDI regions; and 2010 to 2018 in high-SDI regions (APC = 0.0923). Declines occurred during other periods. From 2019 to 2021, declining trends slowed in low-SDI, low-middle-SDI, and high-middle-SDI regions, with APCs of −0.1603, −0.1043, and −0.2010, respectively (Fig. 3).

**Figure 3.**
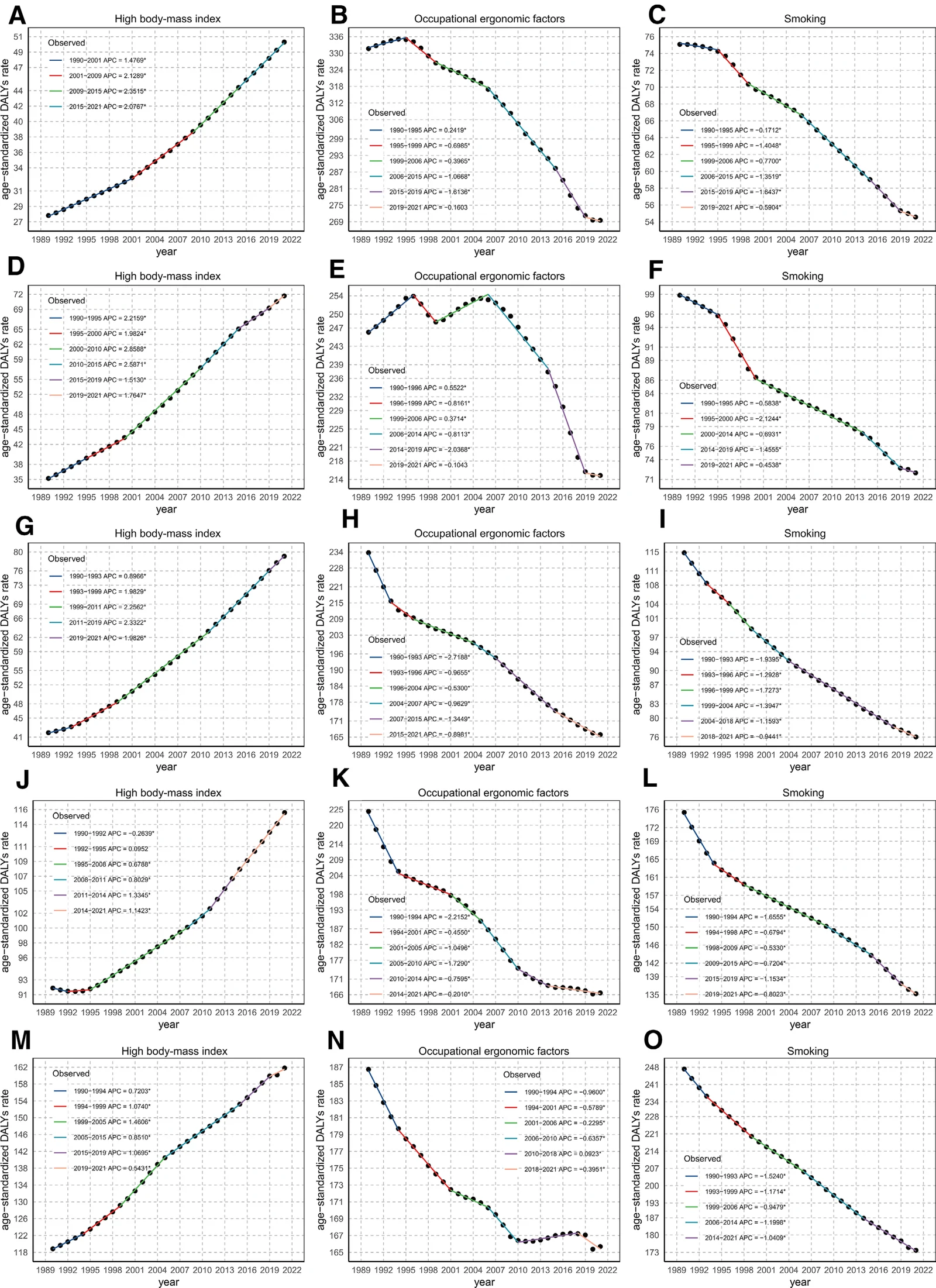
SDI-specific annual percentage change (APC) and trends in global low back pain attributable to low SDI (panels A–C), low-middle SDI (panels D–F), middle SDI (panels G–I), high-middle SDI (panels J–L), and high SDI (panels M–O), with age-standardized DALY rates from 1990 to 2021. APC = annual percentage change, SDI = sociodemographic index.

### 3.2. LBP DALY burden attributable to risk factors across 21 GBD regions

In 2021, East Asia had the highest number of high-BMI-attributable DALYs (1.11 million; 95% UI: 114,372–2.32 million), whereas high-income North America had the highest age-standardized DALY rate (232.48 per 100,000; 95% UI: 23.41–469.44). For occupational ergonomic factors, East Asia had the highest DALYs (3.45 million; 95% UI: 2.38–4.70 million), while Eastern Sub-Saharan Africa had the highest ASR (342.78 per 100,000; 95% UI: 243.97–463.37). East Asia also had the highest smoking-attributable DALYs (1.93 million; 95% UI: 1.13–2.82 million), whereas Central Europe had the highest smoking-attributable age-standardized DALY rate (287.98 per 100,000; 95% UI: 172.36–428.51).

High-BMI-attributable DALYs increased across all 21 GBD regions. The largest increases occurred in South Asia (AAPC = 2.79; 95% CI: 2.74–2.84) and Central Sub-Saharan Africa (AAPC = 2.77; 95% CI: 2.74–2.81), whereas Central Asia had the smallest increase (AAPC = 0.77; 95% CI: 0.76–0.78). Region-specific joinpoint trends in age-standardized DALY rates attributable to high BMI are presented in Figure S1, Supplemental Digital Content 3.

Occupational ergonomic factors contributed to declining LBP burden in most GBD regions, with the largest decrease in East Asia (AAPC = −1.43; 95% CI: −1.51 –−1.35). Conversely, Oceania had the largest increase (AAPC = 1.07; 95% CI: 1.00–1.15) (Table 1). Detailed joinpoint trends in age-standardized DALY rates attributable to occupational ergonomic factors are shown in Figure S2, Supplemental Digital Content 4. Smoking-attributable DALYs declined in most of the 21 GBD regions, except Eastern Europe, where they increased (AAPC = 0.18; 95% CI: 0.13–0.23). The largest declines occurred in Central Latin America (AAPC = −1.79; 95% CI: −1.84– − 1.75) and Southern Sub-Saharan Africa (AAPC = −1.62; 95% CI: −1.65 – − 1.58). Region-specific joinpoint trends in age-standardized DALY rates attributable to smoking are presented in Figure S3, Supplemental Digital Content 5.

Overall, East Asia had the largest absolute DALY burden attributable to all 3 risk factors in 2021. However, the regions with the highest ASRs varied by risk factor: high-income North America for high BMI, Eastern Sub-Saharan Africa for occupational ergonomic factors, and Central Europe for smoking. South Asia and Central Sub-Saharan Africa had the fastest increases in high-BMI-attributable burden. East Asia had the largest decline in occupational ergonomic burden, whereas Oceania had the largest increase. Smoking-attributable age-standardized DALY rates declined in most GBD regions, with Eastern Europe being the only region showing a significant increase (Tables S1–S3, Supplemental Digital Content 1**).**

### 3.3. DALY burden of LBP attributable to risk factors across 204 countries

In 2021, most countries showed increasing trends in high-BMI-attributable burden, whereas occupational ergonomic and smoking-attributable burdens generally declined (Fig. 4). Hungary, Montenegro, and the United States had the highest age-standardized DALY rates attributable to high BMI, at 280.08 (95% UI: 27.41–576.96), 253.85 (95% UI: 25.72–528.41), and 240.98 (95% UI: 24.29–485.99) per 100,000, respectively (Table S4, Supplemental Digital Content 6). For occupational ergonomic factors, Mozambique, Albania, and Bhutan had the highest rates, at 486.96 (95% UI: 350.75–661.84), 452.41 (95% UI: 312.07–638.20), and 442.93 (95% UI: 313.81–609.95), respectively (Table S5, Supplemental Digital Content 7). For smoking, Montenegro, Serbia, and Bosnia and Herzegovina had the highest rates, at 360.29 (95% UI: 213.02–535.14), 337.87 (95% UI: 200.81–501.04), and 323.92 (95% UI: 191.27–491.88), respectively (Table S6, Supplemental Digital Content 8).

**Figure 4.**
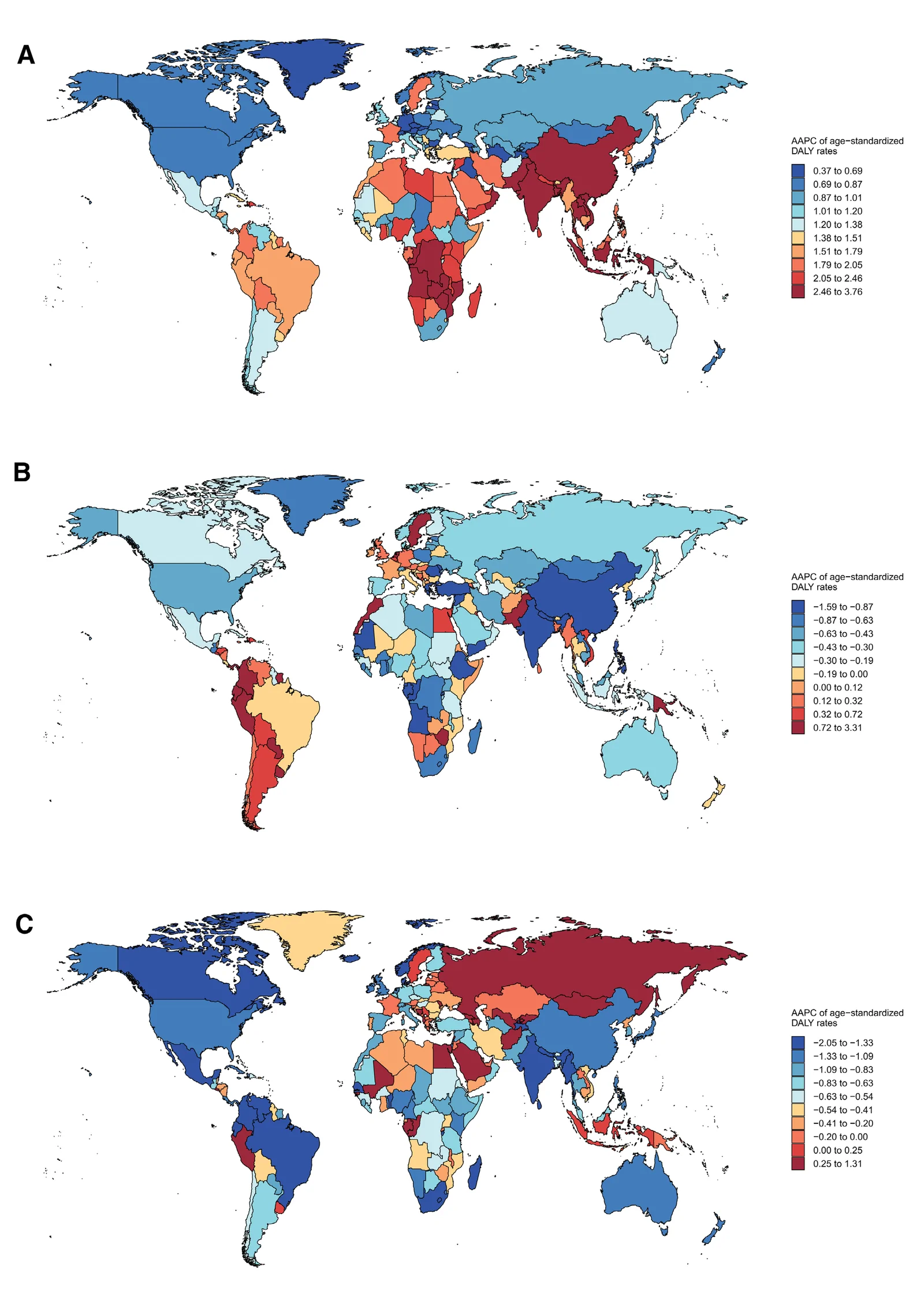
Average annual percentage change (AAPC) in age-standardized DALY rates for low back pain across 204 countries and territories for high body mass index (A), occupational ergonomic factors (B), and smoking (C), 1990 to 2021. AAPC = average annual percentage change, DALYs = disability-adjusted life years.

From 1990 to 2021, Bangladesh (AAPC = 3.76; 95% CI: 3.63–3.89), Zambia (AAPC = 3.37; 95% CI: 3.34–3.41), and Singapore (AAPC = 3.08; 95% CI: 3.02–3.15) had the largest increases in high-BMI-attributable burden (Table S4, Supplemental Digital Content 6). Kiribati (AAPC = 2.57; 95% CI: 2.12–3.02) and Paraguay (AAPC = 1.57; 95% CI: 1.32–1.82) had the largest increases in occupational ergonomic burden. In contrast, Ethiopia (AAPC = −1.59; 95% CI: −1.74–−1.44) and Moldova (AAPC = −1.51; 95% CI: −1.66–−1.37) had the largest declines (Table S5, Supplemental Digital Content 7).

For smoking-attributable LBP, South Africa and Madagascar had the largest reductions, with AAPCs of −1.94 (95% CI: −1.99–−1.90) and −2.05 (95% CI: −2.09–−2.01), respectively. Conversely, Uzbekistan (AAPC = 0.70; 95% CI: 0.66–0.74) and Mali (AAPC = 1.00; 95% CI: 0.96–1.04) had the largest increases (Table S6, Supplemental Digital Content 8).

At the national level, the countries with the highest age-standardized DALY rates in 2021 varied by risk factor. Hungary, Montenegro, and the United States had the highest rates attributable to high BMI; Mozambique, Albania, and Bhutan had the highest occupational ergonomic rates; and Montenegro, Serbia, and Bosnia and Herzegovina had the highest smoking-attributable rates. The largest increases occurred in Bangladesh, Zambia, and Singapore for high BMI; Kiribati and Paraguay for occupational ergonomic factors; and Mali and Uzbekistan for smoking. Ethiopia and Moldova had the largest declines in occupational ergonomic burden, whereas Madagascar and South Africa had the largest reductions in smoking-attributable burden (Tables S4–S6, Supplemental Digital Content 6).

### 3.4. Risk factors of LBP in different sex and age groups

The proportion of health impacts attributable to high BMI increased significantly from 1990 to 2021. Occupational ergonomic factors primarily affected the 15 to 29-year age group, whereas obesity began affecting the 20 to 24-year group and smoking the 30 to 34-year group (Fig. 5). Individuals aged ≥85 years were primarily affected by high BMI and smoking. In 2021, the 50 to 54-year age group had the highest DALY burden across all 3 risk factors (Fig. 6). However, high-BMI-attributable DALYs peaked among females aged 70 to 74 years; occupational ergonomic factors peaked at 50 to 60 years; and smoking-attributable DALYs peaked at 60 to 69 years (Fig. 6). Females had higher BMI- and occupation-attributable DALYs, whereas males had higher smoking-attributable burdens.

**Figure 5.**
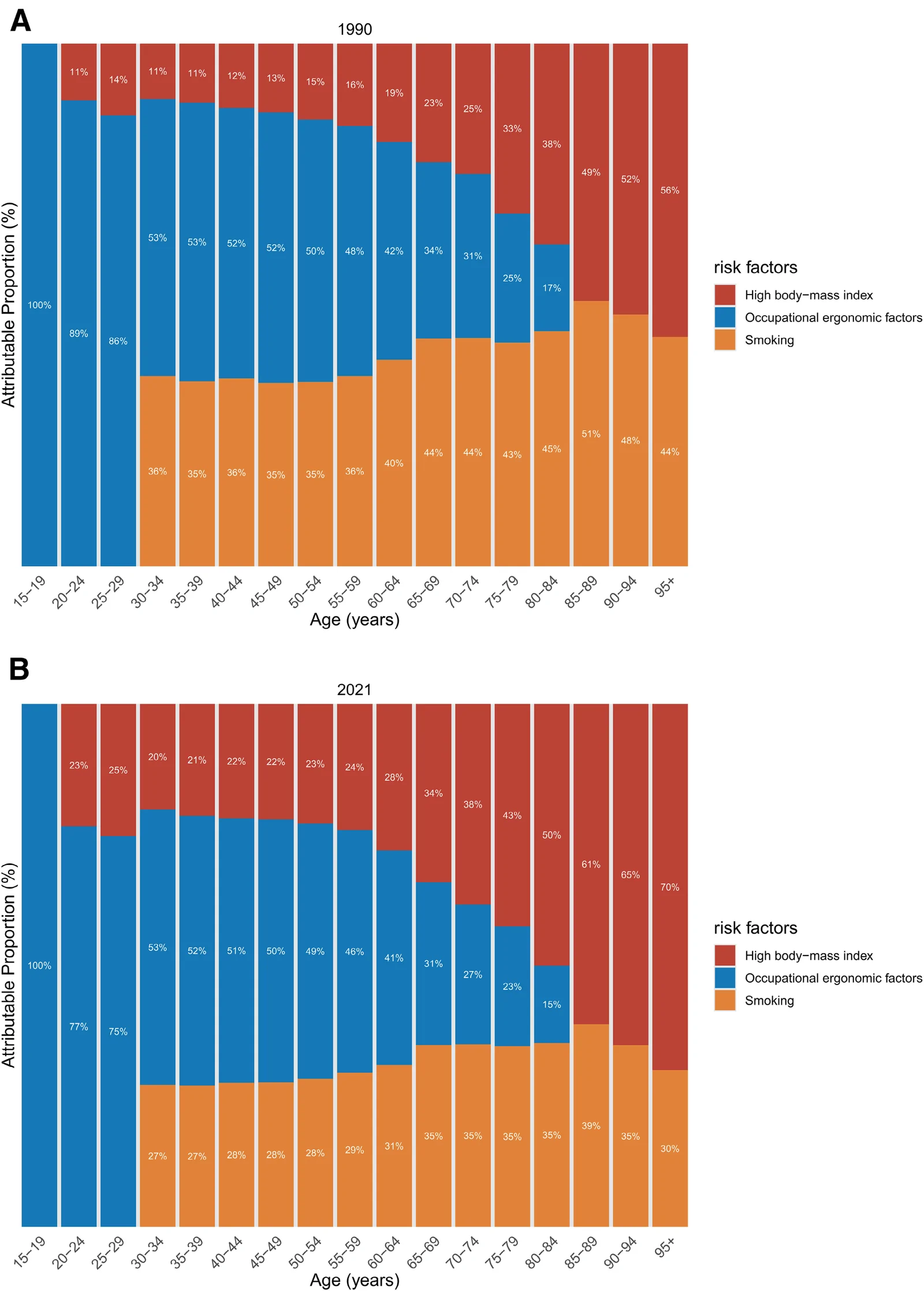
Age-specific proportions of DALYs attributable to risk factors for global low back pain in 1990 (A) and 2021 (B). DALYs = disability-adjusted life years.

**Figure 6.**
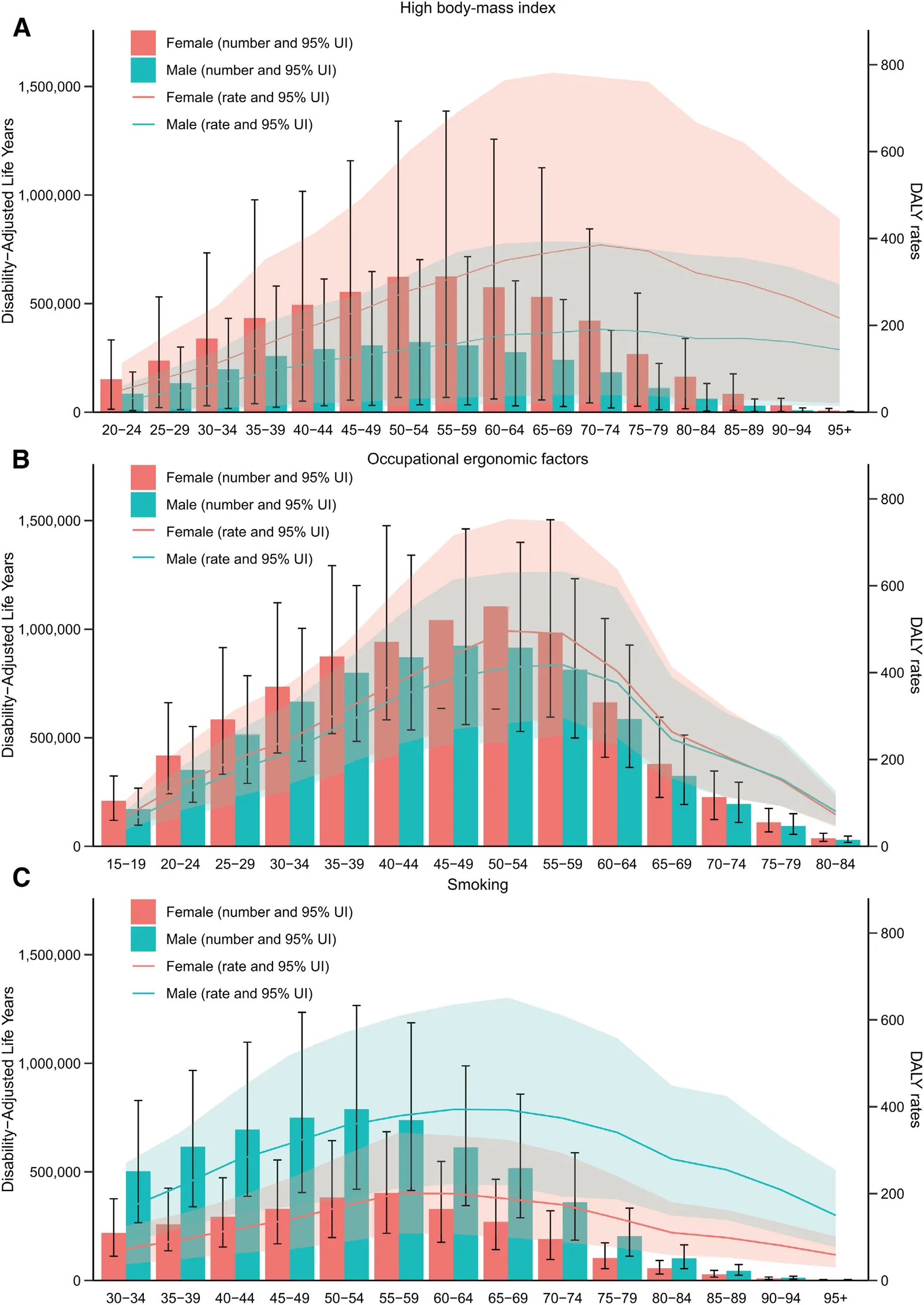
Age-specific numbers and rates of DALYs of low back pain attributable to 3 risk factors by sex in 2021: high body mass index (A), occupational ergonomic factors (B), and smoking (C). DALYs = disability-adjusted life years, UI = uncertainty interval.

### 3.5. Correlation between SDI and age-standardized DALY rates attributable to risk factors

Age-standardized DALY rates attributable to high BMI were strongly and positively correlated with SDI (*R* = 0.671, 95% CI: 0.627–0.710, *P* < .001) (Fig. 7). Smoking also showed a strong positive correlation with SDI (*R* = 0.721, 95% CI: 0.683–0.756, *P* < .001), indicating higher smoking-attributable rates in countries with higher SDI (Fig. 7). In contrast, occupational ergonomic factors were inversely correlated with SDI (*r* = −0.414, 95% CI: −0.475–−0.349, *P* < .001), indicating a higher occupational ergonomic burden in countries with lower SDI (Fig. 7).

**Figure 7.**
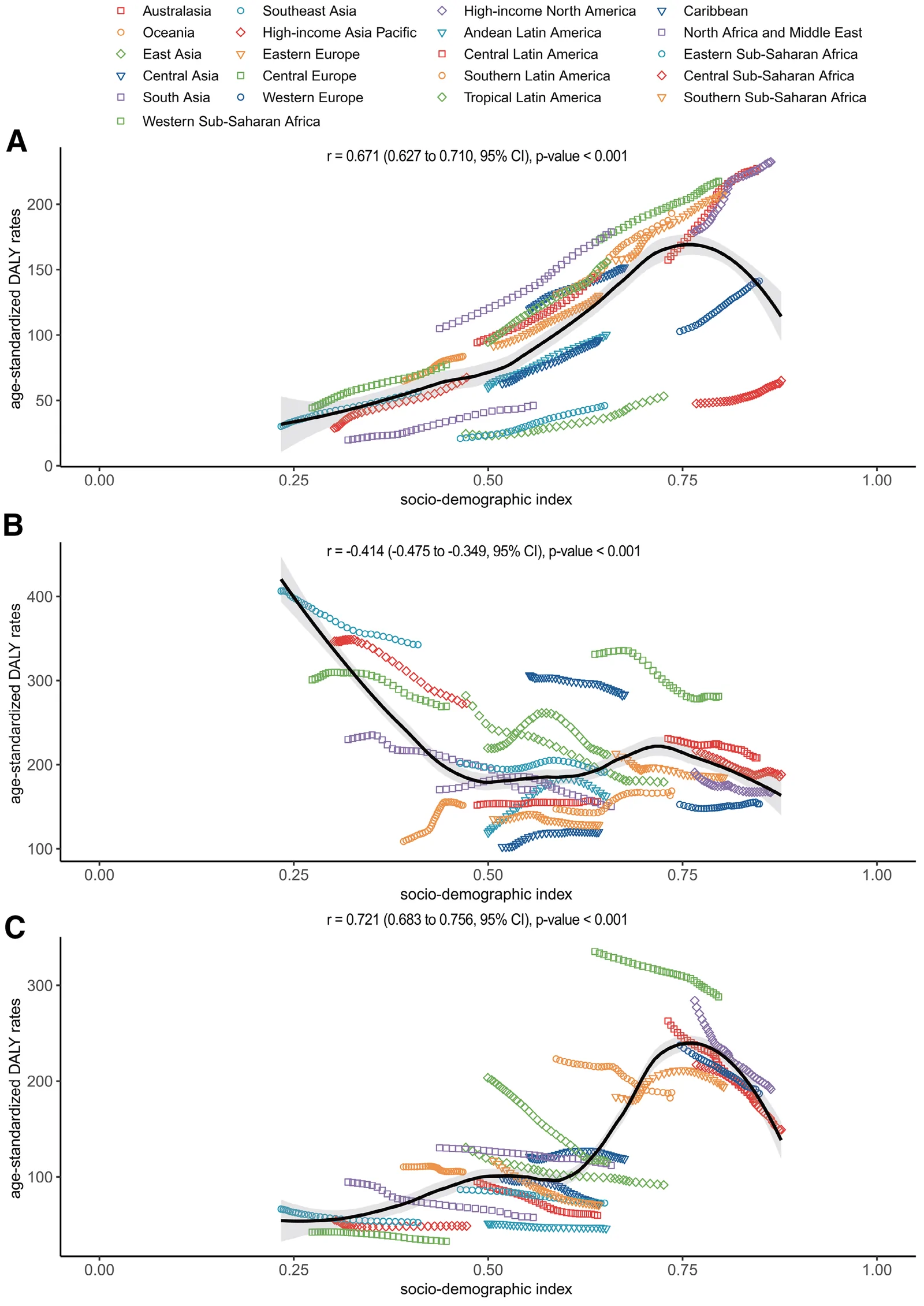
Associations between age-standardized DALY rates and sociodemographic index for low back pain attributable to high body mass index (A), occupational ergonomic factors (B), and smoking (C) across 21 GBD regions, 1990–2021. CI = confidence interval, DALYs = disability-adjusted life years.

## 4. Discussion

The GBD findings reveal substantial regional variation in LBP burden attributable to high BMI, occupational ergonomic factors, and smoking. These patterns reflect differences in the epidemiology of LBP and the socioeconomic conditions that shape its distribution and burden.

### 4.1. Increasing burden associated with high BMI

Globally, rising high BMI prevalence has increased associated mortality and DALYs from cardiovascular disease, diabetes, kidney disease, and LBP.^[22]^ LBP ranks sixth among the causes of DALYs attributable to high BMI.^[23]^ The association between obesity and LBP may involve mechanical stress on spinal structures and systemic inflammation, both of which can exacerbate pain and disability.^[24,25]^

From 1990 to 2021, high-BMI-attributable DALYs increased from 3.09 million to 8.36 million, paralleling the global increase in obesity and LBP incidence. East Asia had the highest number of high-BMI-attributable DALYs (1.11 million; 95% UI: 114,372–2.32 million), whereas high-income North America had the highest age-standardized DALY rate (232.48 per 100,000; 95% UI: 23.41–469.44). Low-middle-SDI regions experienced the fastest increase in high-BMI-attributable LBP burden (AAPC = 2.32; 95% CI: 2.31–2.34). Bangladesh and Zambia had particularly high AAPCs of 3.76 and 3.37, respectively, underscoring the need for interventions to address the growing burden of high-BMI-attributable LBP.^[26]^

High-SDI regions also experienced a substantial high-BMI-attributable LBP burden.^[27]^ In 2021, Hungary, Montenegro, and the United States had among the highest age-standardized DALY rates. These population-level differences may reflect variations in high BMI prevalence, age structure, lifestyle, and healthcare systems. However, these factors were not directly measured, and their contributions to regional differences cannot be determined from the present analysis.

The rapid increase in low-middle-SDI regions may reflect the growing importance of high BMI during demographic, socioeconomic, and epidemiological transitions.^[28,29]^ Because this study used aggregate GBD estimates, these associations should not be interpreted as evidence that specific behavioral, genetic, sociocultural, or healthcare factors caused regional or national differences.^[26]^

These findings support context-specific strategies promoting healthy weight, physical activity, and access to preventive and weight-management services.^[30]^ Individual-level and longitudinal studies are needed to clarify mechanisms underlying geographic disparities and evaluate intervention effectiveness.

### 4.2. Occupational ergonomics: changing trends and implications

Globally, DALYs attributable to occupational ergonomic factors increased from 10.85 million to 15.57 million, whereas the age-standardized DALY rate declined. This divergence suggests that demographic changes, including population growth and aging, may have contributed to the increase in absolute burden despite a reduction in the population-standardized rate. However, the present ecological analysis cannot determine the specific factors responsible for these temporal trends.^[8]^ Temporal patterns also differed by SDI level. In low- and middle-SDI settings, occupational ergonomic burden increased during several periods, whereas a modest increase was observed in high-SDI regions during 2010 to 2018. From 2019 to 2021, the declining trends slowed in several SDI groups. These heterogeneous patterns may reflect differences in occupational structures and socioeconomic conditions across regions.^[31,32]^ The occupational ergonomic burden peaked in middle age and declined in older age groups. Reduced occupational exposure after retirement may partly contribute to this pattern, although individual employment status and occupational history were not available in the GBD data used in this analysis.^[33]^ Sex differences were also observed, with variation across SDI groups. Such differences may be related to sex-specific occupational and social exposures reported in previous studies.^[34]^ These findings support the continued importance of occupational health strategies tailored to local working conditions. Future intervention studies should evaluate which ergonomic approaches are effective and feasible across different socioeconomic and occupational settings.

### 4.3. Smoking: A dual-faceted risk factor

Smoking-attributable LBP DALYs increased from 6.78 million to 8.82 million, whereas the ASR declined. The divergence between absolute and age-standardized burden is consistent with the influence of population growth and aging. Regional differences were substantial, although the present analysis cannot determine the specific behavioral, socioeconomic, or policy factors underlying these patterns. Smoking-attributable age-standardized DALY rates declined in most GBD regions, with the largest decreases observed in Central Latin America and Southern Sub-Saharan Africa. Eastern Europe was the only region with a significant increasing trend (AAPC = 0.18), and Montenegro, Serbia, and Bosnia and Herzegovina had the highest ASRs in 2021. These findings demonstrate substantial geographic heterogeneity in smoking-attributable LBP burden. Although differences in smoking prevalence and tobacco-control environments may contribute to these patterns, such factors were not directly evaluated in the present study.^[35–37]^ Variation in smoking prevalence and tobacco-control environments may partly underlie these regional differences. Smoking-attributable LBP disproportionately affects males. In 2019, global DALYs among males were nearly twice those among females (6.76 million vs 3.21 million).^[38]^ The occupational ergonomic burden peaked in middle age and declined thereafter, a pattern that may partly reflect reduced occupational exposure at older ages.^[39]^

### 4.4. Limitations

This study evaluated the contribution of 3 risk factors to LBP burden using the latest GBD 2021 data. This study has several limitations. First, GBD estimates are model-based and may be less precise in regions with limited primary epidemiological data, particularly low-income countries in Sub-Saharan Africa. Second, the analysis included only 3 risk–outcome pairs available within the GBD 2021 framework: high BMI, occupational ergonomic factors, and smoking. Other relevant factors, including psychosocial stress, physical inactivity, genetic susceptibility, comorbidities, and healthcare access, were not assessed. Therefore, the findings do not represent the full etiological spectrum of LBP and should not be used to rank all potential risk factors by importance. Third, the ecological design precludes causal inference. For example, the association between high BMI and LBP may reflect shared pathways (e.g., inflammation) rather than direct causation. Fourth, occupational ergonomic factors were broadly aggregated, potentially obscuring differences among specific exposures (e.g., sedentary work vs manual labor). Fifth, smoking-related DALYs did not distinguish active from passive smoking or account for emerging nicotine products (e.g., vaping).

## 5. Conclusion

In summary, the global LBP burden attributable to high BMI, occupational ergonomic factors, and smoking remains substantial and varies considerably across regions, age groups, sexes, and levels of socioeconomic development. The increasing burden attributable to high BMI warrants particular attention, while occupational ergonomic factors remain the largest contributor among the 3 GBD-attributable risks examined. These findings may help inform risk-specific prevention priorities, although the effectiveness of particular interventions should be evaluated in longitudinal and intervention studies.

## Acknowledgments

The contributions of the GBD 2021 Risk Factors Collaborators are gratefully acknowledged.

## Author contributions

**Conceptualization:** Yulan Ba.

**Data curation:** Yulan Ba, Xuetao Yu.

**Formal analysis:** Yulan Ba, Xuetao Yu.

**Methodology:** Yulan Ba.

**Resources:** Yulan Ba.

**Validation:** Yulan Ba.

**Supervision:** Jinxian Li.

**Writing – original draft:** Yulan Ba, Xuetao Yu, Erman Wu.

**Writing – review & editing:** Yulan Ba, Erman Wu, Jinxian Li.


















